# Histological and Inflammatory Changes in Thyroid Glands and Early Growth Outcomes in Offspring of Rats Exposed to Ambient Air Pollution near a Petrochemical Complex: A Preliminary Study

**DOI:** 10.3390/life16020329

**Published:** 2026-02-13

**Authors:** Maria Angela Zaccarelli-Marino, Nuha A. Dsouki, Rodrigo P. de Carvalho, Juliana M. Veridiano, Monica A. Sato

**Affiliations:** 1 Internal Medicine Department, ABC Medical School Foundation, Santo André 09060-870, SP, Brazil; 2 Laboratory of Molecular and Translational Endocrinology, Department of Medicine, Escola Paulista de Medicina, Federal University of São Paulo, São Paulo 04022-001, SP, Brazil; 3 Department of Morphology and Physiology, Centro Universitário FMABC, Santo André 09060-870, SP, Brazil; r.pigozzi@hotmail.com (R.P.d.C.);

**Keywords:** histology, thyroid gland, body weight, craniocaudal length, offspring rats, pollution, industrial

## Abstract

Background: Environmental agents can disrupt thyroid function at several levels, including the synthesis, action, and excretion of thyroid hormones, and an inadequate concentration of thyroid hormones affects almost all organs and systems. Objective: The present study aimed to evaluate the histology and presence of the cytokines TNFα, IL-6, and IL-10 in the thyroid gland by immunohistochemical labeling, as well as the body weight and craniocaudal length of pups of Wistar rats exposed to ambient air in the vicinity of the Capuava Petrochemical Complex (CPC), located in the Santo André and Mauá cities, at São Paulo State, Brazil. Methods: This study used Wistar rats between 14 and 16 weeks of age, distributed in couples, that were exposed to pollution from the CPC located in the regions of Santo André and Mauá, Sao Paulo State, Brazil. One couple was positioned 600 m (SS1), and another was 1000 m (SS2) from the CPC, while the control group was kept at the animal research facility of the Physiology Laboratory of the FMABC University Center, Santo André. After mating, the resulting offspring were monitored for four weeks, with their body weight and craniocaudal length measured weekly. Subsequently, the offspring’s thyroid glands were histologically analyzed using H&E staining and immunohistochemistry to detect the presence of inflammatory cytokines (TNF-α, IL-6, and IL-10). Results: In the SS1 group, the thyroid glands showed follicular heterogeneity with macrofollicles and numerous microfollicles without colloid, lined by flattened epithelial cells. In these thyroid follicles, there was intense TNFα (*p* = 0.002) staining, slight IL-6 staining (*p* = 0.042), and significantly stronger staining for IL-10 (*p* = 0.013) compared to that in the control group. This group also had a significantly lower body weight than the control animals on the 6th, 13th, and 20th days of life. In the SS2 group, the thyroids presented an architecture dominated by microfollicles without colloid as well as inflammatory cells in the colloid of some follicles. Immunohistochemistry revealed intense pan-follicular TNFα (*p* = 0.002) staining, with additional cytoplasmic staining of IL-6 (*p* = 0.040) and IL-10 (*p* = 0.006). The SS2 group also showed a more pronounced deficit compared to the SS1 group in terms of birth weight. The cranial–caudal length was shorter on the 13th and 20th days of life in the SS1 and SS2 groups compared to the control group. Conclusions: The results indicate that proximity of rats to the CPC was a determining factor in the development of histological abnormalities and increases in inflammatory cytokine markers in the thyroid glands of the offspring. In addition, the offspring born near the CPC had lower birth weights and shorter craniocaudal lengths compared to the animals in the control group.

## 1. Introduction

The state of São Paulo (SP) is the most populous and industrialized in Brazil, with a population of around 45 million [1] and 7012 petrochemical plants [2].

The Metropolitan Area of Sao Paulo (MetASP) is situated in SP in southeast Brazil, and covers an area of 8000 km^2^, has a population of 21.9 million people, and is considered the fifth largest urban conglomeration in the world [3]. The area houses industrial complexes, which produce around 30% of fuels by volume and represent the largest consumer market in South America [4].

The MetASP comprises seven major cities, known collectively as the ABC region, with a population of 2.8 million people. The Capuava Petrochemical Complex (CPC), which is deemed the most important industrial complex in the ABC region that contributes to poor air quality, is located within Santo Andre and Maua cities [5]. This complex produces gasoline, diesel (S-10), liquefied petroleum gas (LPG), ethylene, propylene, and polyethylene, which are derived from distilling naphtha, as well as fertilizers and many intermediates. It can process over 53,000 barrels of oil per day [4].

Refineries and petrochemical facilities release volatile organic compounds (VOCs), particulate material (PM), and greenhouse gases as a byproduct of various operations [6], and these can have potential deleterious effects on water, soil, and the air. Although these activities are an important source of energy and economic development, they can pollute the environment, with consequent negative health impacts for humans and animals [7]. Hydrocarbons, both aliphatic and aromatic, are the main VOCs produced by the oil industry [8]. In 2012, the World Health Organization (WHO) estimated the number of VOC-related deaths to be 6.5 million, accounting for 11.6% of all deaths related to atmospheric pollution and over 600 million people worldwide affected by its acute and chronic effects [9].

The population of the MetASP is exposed to high levels of pollutants from petroleum [10], including a host of organic compounds that pose a hazard to human health and the environment. Ozone (O_3_) and fine PM (PM 2.5) are the pollutants that often exceed limits for state air quality standard regulations. Evidence indicates that the population living in the vicinity of the CPC exhibits a higher prevalence of autoimmune thyroid diseases (AITDs) [11,12] and primary hypothyroidism (PH) [13,14]. Benvenga et al. [15] have also observed thyroid disease in other industrial regions across the globe.

A study has indicated that air pollution can cause prenatal immediate and long-term health effects [16], particularly those involving pregnancy and birth cohorts. It has been reported that air pollution may impact thyroid function [17,18,19,20,21]. Immune response, oxidative stress and inflammation are critical mechanisms in the transduction of systemic toxicity associated with air pollution exposure [22]. Industrial activities have been implicated as the main source of heavy metal con-tamination [23]. Some studies also suggest that exposure to heavy metals can disrupt reproductive function and may adversely affect embryo development [24,25]. In addition, a previous report by Rana [25] demonstrated that the kinetics of thyroid hormones are affected by different heavy metals. Petroleum-derived emissions frequently contain heavy metals such as vanadi-um (V) and nickel (Ni) [16]. These elements can cross the placental barrier, accumulate fetal tissues, and act as endocrine disruptors capable of affecting placental and fetal de-velopment [24,26,27]. Endocrine-disrupting compounds interfere with hormone synthesis, secretion, transport, metabolism, or receptor action, compromising physiologi-cal homeostasis and developmental processes [28]. Environmental agents can disrupt thyroid function at multiple levels, and altered thyroid hormone concentrations affect roughly all organ systems [29,30]. Experimental animal models have been extensively used to investigate pollutant toxicity and have demonstrated structural, metabolic, endocrine, and immunological dis-turbances following exposure [31,32,33]. Studies examining individual contami-nants report developmental delay, fetal malformations, inflammatory responses, and neu-rotoxicity [34,35,36,37]. Disturbances in zinc (Zn) and copper (Cu) metabolism were also found by Borowska et al. [37] in their study involving rats exposed to cadmium (Cd). Their findings demonstrated that, after treating female Wistar rats with 1 mg/kg of Cd for 10 months, the absorption of Zn declined, but following exposure to 5 mg/kg of Cd, the absorption of Zn increased after 3 and 24 months. Nevertheless, most ex-perimental approaches evaluate isolated chemical agents under controlled laboratory con-ditions and thereby may not reflect real-world exposure scenarios. In natural environ-ments, populations living near petrochemical complexes are chronically exposed to com-plex mixtures of airborne pollutants rather than single compounds. Although intrauterine exposure to ambient air pollution has been associated with impaired fetal growth and endocrine disruption, populations living near petrochemical complexes are chronically exposed to complex mixtures of airborne pollutants rather than isolated compounds, and the developmental health consequences of such real-world mixed chemical exposures remain insufficiently understood. Clarifying this gap is important for strengthening translational relevance in environmental health research. Thus, the present study aimed to evaluate the histology and presence of the cytokines TNFα, IL-6, and IL-10 in the thyroid gland by immunohistochemical labeling, as well as the body weight and craniocaudal length of pups of Wistar rats exposed to ambient air in the vicinity of the CPC located in Santo André and Mauá cities, at São Paulo State, Brazil.

## 2. Materials and Methods

### 2.1. Sampling Site

**Figure 1:**
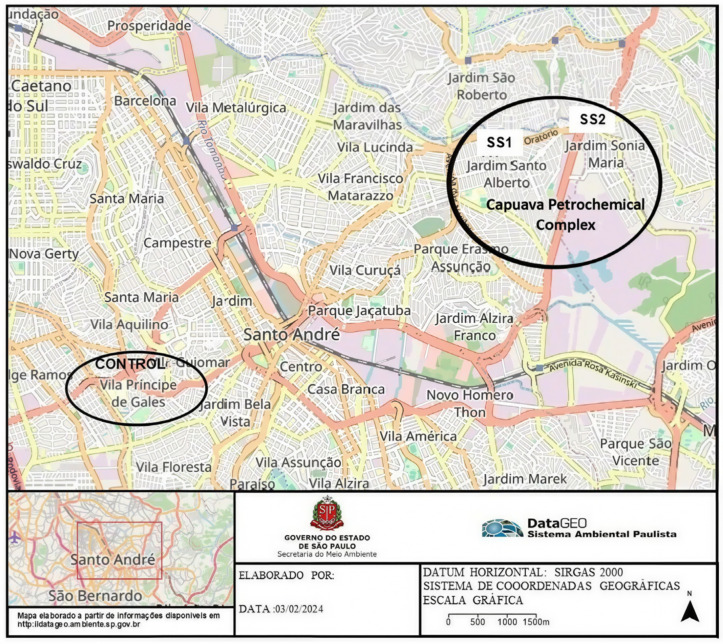
Map of cities of Santo André and Mauá depicting location of exposure and control groups of animals: sampling site 1 (SS1) at Rua Petrogrado, Jardim Santo Alberto, Santo André, SP, located approximately 600 m from CPC; sampling site 2 (SS2) at Rua Noel Rosa, Jardim Sonia Maria, Mauá, SP, located roughly 1000 m from CPC; control group, located at animal research facility of Physiology Laboratory at Centro Universitário FMABC, Vila Sacadura Cabral/Vila Príncipe de Gales district, Santo André, SP, ~7500 m from CPC (http://datageo.ambiente.sp.gov.br, accessed on 2 February 2024).

Sampling was performed in the city of Santo André, SP, at Rua Petrogrado, Jardim Santo Alberto, located roughly 600 m from the CPC (SS1), and in the city of Mauá, SP, at Rua Noel Rosa, Jardim Sonia Maria, located approximately 1000 m from the CPC (SS2), on the southeast border of the MetASP, SP, Brazil. The control group was located at the animal research facility of the Physiology Laboratory at Centro Universitário FMABC, Vila Sacadura Cabral/Vila Príncipe de Gales district, Santo Andre, SP, ~7500 m from the CPC (http://datageo.ambiente.sp.gov.br, accessed on 2 February 2024; see Figure 1).

The CPC is located in Parque Capuava in the city of Santo André, SP, Brazil, and began operations in the mid-1950s, leading to a rapid increase in the population density of the surrounding area. In 2022, Santo André City had a population of 748,919; meanwhile, in 2022, Mauá City had a population of 418,261. According to the last census of 2022, the population density was 4260 inhabitants/km^2^ in Santo André City, and 6753 inhabitants/km^2^ in Mauá City, SP [38].

### 2.2. Animals

Heterogeneous Wistar rats (14–16 weeks-old, *n* = 6)—3 males (~350 g) and 3 females (~270 g) supplied from Animal Research facility of the Centro Universitário Faculdade de Medicina do ABC (FMABC), Santo Andre/Brazil—were used. The animals were fed with standard pellets (Nuvilab^®^) and water ad libitum. All study procedures were approved by the Animal Ethics Committee of the Centro Universitário Faculdade de Medicina do ABC (CEUA-FMABC) (protocol number 14/2018).

**Figure 2:**
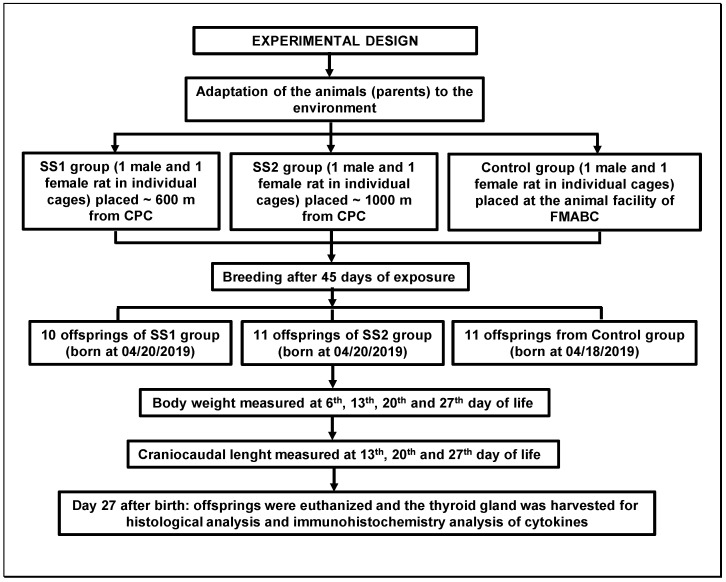
Flowchart showing the experimental design details.

In January 2019, the animals were divided into 3 groups, each containing 1 male and 1 female, which were placed at sampling site 1 (SS1, *n* = 2) or sampling site 2 (SS2, *n* = 2), or kept in the lab as a control group (*n* = 2) (Figure 2).

The couples at SS1 and SS2 were housed in an area external to the residences. Prior to breeding, the animals were kept in individual stainless steel cages, surrounded by a second protective external grid 10 cm from the cage, preventing encroachment by other invasive animals or predators. Maintenance and cleaning of cages took place twice a week, and 250 g of chow pellets was supplied to the animals as well as 320 mL of filtered water in drinking bottles. Each time the cages were cleaned, leftover feed and water in drinking bottles were collected to assess intake of the exposed and control animals.

Throughout the experiment, the control group animals (1 male and 1 female) were kept at the animal research facility of the Physiology Laboratory (FMABC), located ~7500 m from the CPC (Figure 1) in stainless steel cages on a ventilated shelf (Alesco^®^, Monte Mor, São Paulo, Brazil) with air filtered by 3 EPA filters. A 12 h dark/12 h light cycle was maintained. The room temperature was kept at ~22 °C and relative air humidity at ~60%.

After 45 days of adaptation to the environment, for each group (SS1, SS2, and control), the rats were placed in the same cage for breeding and produced the following offspring: SS1 (*n* = 10), SS2 (*n* = 11), and control (*n* = 11). At birth, body weight and craniocaudal length were measured, and weekly follow-ups were carried out for 4 weeks.

After this environmental exposure period, the adult animals (males and females of SS1, SS2, and control groups), together with their respective descendants, were euthanized at the Physiology Laboratory of the FMABC using an overdose of 4% isoflurane in 100% O_2_. Subsequently, the thyroid glands of the offspring were harvested and fixed in 10% buffered formalin solution for at least 24 h for later histological and immunohistochemistry analyses.

### 2.3. Morphological Analysis

#### 2.3.1. Histological Sections

After the thyroid glands had been kept in 10% formalin for at least 24 h for fixation, the samples were rinsed in distilled water and dehydrated in a series of increasing concentrations of ethanol (70°, 80°, 95°, and 100°). The samples were kept in each ethanol concentration for 30 min. Afterwards, they were submitted to diaphonization using 100% xylol solution for 15 min, a process repeated in triplicate. After these processes, the samples were bathed in paraffin at 65 °C twice for 1 h and then embedded into paraffin blocks.

The paraffin blocks were cut into 5 µm thick sections using a LEICA—RM 2245 manual microtome (Leica, Nussloch, Germany). The histological sections were mounted on slides and stained with Hematoxylin–Eosin (H&E) for morphological assessments and immunohistochemistry analyses (anti-TNFα, anti-IL-6, and anti-IL-10).

#### 2.3.2. Hematoxylin—Eosin Staining

After deparaffinizing and hydration, the sections were stained using Harris Hematoxylin for 2 min, differentiated in running water for 5 min and then stained using aqueous eosin for 1 min. The sections were then dehydrated in a series of increasing ethanol concentrations (70°, 80°, 95° and 100°), diaphonized in 100% xylol solution, and coverslipped using Entellan^®^ (Merck Group’s, Darmstadt, Germany).

Slides were morphologically analyzed using a vertical Axio Imager M1 Microscope (Zeiss Group, Oberkochen, Germany) and photographed with an AxioCamMRc camera (Zeiss Group, Oberkochen, Germany) at 200× magnification. The images were captured using the capture program AxioVision 4.8, installed on a computer at the Laboratory of Molecular and Translational Endocrinology (LEMT) of the Universidade Federal de São Paulo, UNIFESP, São Paulo City, SP, Brazil.

#### 2.3.3. Immunohistochemistry

The tissue sections were deparaffinized in 100% xylol solution and hydrated in a series of alcohol concentrations (100°, 90° and 70°), rinsed in distilled water, and subjected to antigen recovery in 10 mM citrate solution at pH 6.0 and 100 °C. After cooling, endogenous peroxidase blocking and blocking of non-specific sites was performed for 10 min using the specific mouse and rabbit kit HRP/DAB IHC Detection-Micro-polymer Abcam (ab236466). The tissue samples were immersed in PBS 1x buffer solution at pH 7.2 for rinsing and then incubated with primary antibodies at 4 °C for 14–16 h.

The primary antibodies used were anti-IL-6 from Santas Cruz Biotechnology (Dallas, TX, USA) (SC-130326), anti-IL-10 from Santas Cruz Biotechnology (Dallas, TX, USA) (SC-7888), anti-TNFα from Cloud-Clone Corp. (Katy, TX, USA) (PAA133Ra01), diluted at 1:100 in 1% BSA. After incubation with the primary antibodies, the tissue samples were rinsed in PBS 1x buffer and incubated at room temperature for 10 min with mouse-specifying reagent and then for 15 min with goat anti-rabbit HRP conjugate (ab236466, Abcam, Waltham, MA, USA). The secondary system was bound to the primary antibody in accordance with the manufacturer’s suggested protocol.

Afterwards, the tissue specimens were exposed for 1 min in 3,3-diaminobenzidine solution (DAB) (ab236466, Abcam, MA, USA) and counterstained with Harris Hematoxylin.

Images were acquired using a vertical Axio Imager M1 microscope (Zeiss, Germany) and photographed with an AxioCamMRc camera (Zeiss, Germany) at 200× magnification. The images were captured using the capture program AxioVision 4.8, installed on a computer at the Laboratory of Molecular and Translational Endocrinology (LEMT) of the Universidade Federal de São Paulo, UNIFESP, São Paulo City, SP, Brazil.

#### 2.3.4. Analysis of TNFα, IL6, and IL10 in Thyroid

The quantitative analysis of histological sections allows for the thyroid parenchyma to be evaluated through immunohistochemical techniques (anti-TNFα, anti-IL-6, and anti-IL-10). For each section, four fields of view were randomly photographed and analyzed, highlighting the follicles. Images were captured using a vertical Axio Imager M1 microscope (Zeiss Group, Oberkochen, Germany) and photographed with an AxioCam MR camera (Zeiss Group, Oberkochen, Germany) using the AxioVision 4.8 acquisition software, at 200× magnification. The immunomarkers were quantified using the Color Deconvolution plugin with thresholding in ImageJ version 1.54k (NIH, Bethesda, MD, USA).

### 2.4. Statistical Analysis

The Kolmogorov–Smirnov test was used to evaluate the normality of the data distribution. If the variables exhibited a parametric (normal) distribution, the results are expressed as mean ± S.E.M. One-way analysis of variance (ANOVA) was performed followed by Tukey’s post hoc test to compare body weight, craniocaudal length, and levels of IL-6, Il-10, and TNFα expression in the thyroid gland of the animals exposed at 600 m (SS1 group) and 1000 m (SS2 group) from the CPC, and the control animals. The variables were analyzed using the IBM SPSS Statistics 26 software, and the significance level was set at *p* < 0.05.

## 3. Results

### 3.1. Effect of Environmental Exposure on Offspring Body Weight After Birth

**Figure 3:**
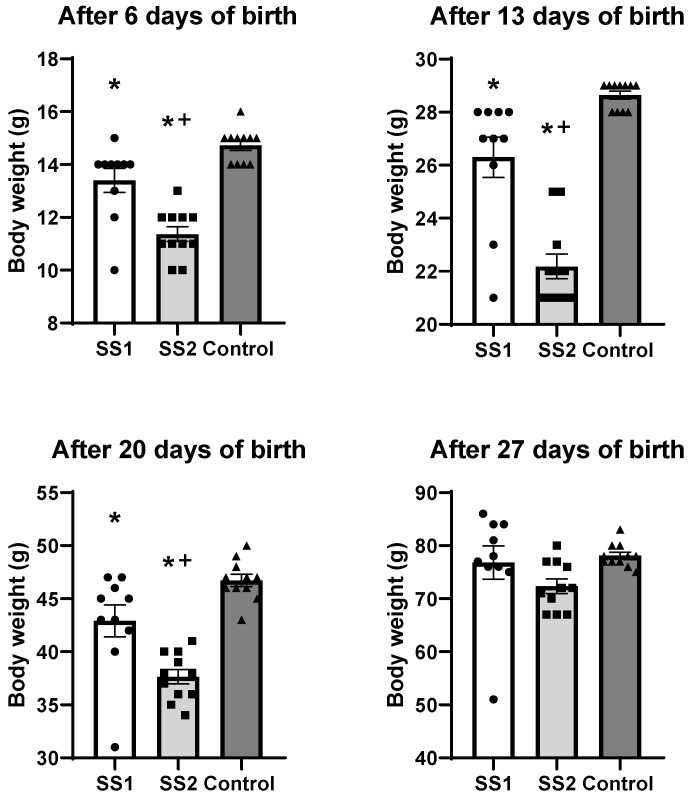
Body weight of offspring (g) at 6, 13, 20, and 27 days of life. Data are presented as mean ± SEM. A significant reduction in body weight was observed in exposed groups (SS1 and SS2) compared to the control group at days 6, 13, and 20 (* *p* < 0.05 vs. control group. + *p* < 0.05). The SS2 group showed significantly lower body weight than SS1 at the same time points (+ *p* < 0.05 vs. SS2 group). No significant differences were observed at day 27. (One-way ANOVA followed by Tukey’s post hoc test).

The descendants of the SS1 group (*n* = 10) had a significantly lower body weight than the control animals (*n* = 11) on the 6th (13 ± 0.45 exposed vs. 15 ± 0.19 g control, *p* = 0.017), 13th (26 ± 1 exposed vs. 29 ± 0.15 g control, *p* = 0.008) and 20th days of life (43 ± 2 exposed vs. 47 ± 1 g control, *p* = 0.025) (Figure 3). Nevertheless, on the 27th day of life, this difference was no longer statistically significant when comparing the exposed (77 ± 3 g) and control group (78 ± 1 g, *p* = 0.885) (Figure 3).

The descendants of the SS2 group (*n* = 11) also showed a significantly reduced body weight on the 6th (11 ± 0.27 exposed vs. 15 ± 0.19 g control, *p* < 0.001), 13th (22 ± 0.46 exposure vs. 29 ± 0.15 g control, *p* < 0.001), and 20th days of life (38 ± 1 exposed vs. 47 ± 1 g control, *p* < 0.001) compared to the control animals (*n* = 11) (Figure 3). However, on the 27th day of life, no significant difference in the SS2 group’s body weight was observed in comparison to the control group (72 ± 1 exposed vs. 78 ± 1, g control, *p* = 0.098) (Figure 3).

When analyzing the SS1 and SS2 groups at different timepoints, the SS2 descendants had lower body weight than the SS1 descendents on the 6th (13 ± 0.45 SS1 vs. 11 ± 0.27 g SS2, *p* < 0.001), 13th (26 ± 1 SS1 vs. 22 ± 0.46 g SS2, *p* < 0.001), and 20th days of life (43 ± 2 SS1 vs. 38 ± 1 g SS2, *p* = 0.002) (Figure 3). No difference was observed in the body weight of the SS1 and SS2 groups on the 27th day of life (77 ± 3 SS1 vs. 72 ± 1 g SS2, *p* = 0.252) (Figure 3).

### 3.2. Effect of Environmental Exposure on Craniocaudal Length of Offspring After Birth

**Figure 4:**
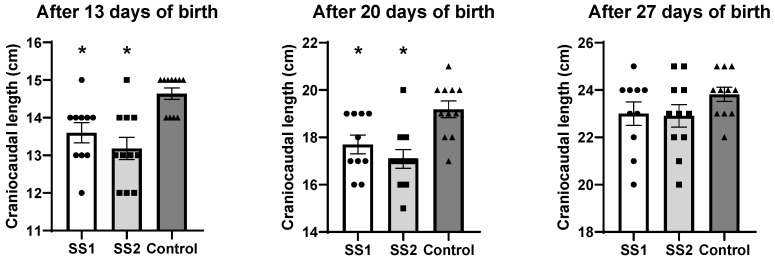
Craniocaudal length of offspring (cm) on 6, 13, 20, and 27 days of life. Data are expressed as mean ± SEM. Significant reductions were detected in exposed groups compared to the control group on days 13 and 20 (* *p* < 0.05), whereas no significant difference was observed on day 27. (One-way ANOVA followed by Tukey’s post hoc test).

In the SS1 group (*n* = 10), the craniocaudal length was lower on the 13th (14 ± 0.26 exposed vs. 15 ± 0.15 cm control, *p* = 0.016) and 20th days of life (18 ± 0.39 exposed vs. 19 ± 0.35 cm control, *p* = 0.028) compared to the control animals (*n* = 11) (Figure 4). In contrast, on the 27th day of life, no significant difference was observed in the craniocaudal length of the SS1 group (23 ± 0.49 cm) compared to the control animals (24 ± 0.29 cm, *p* = 0.385) (Figure 4).

Similarly, in the SS2 group, the craniocaudal length of the descendants of the exposed groups (*n* = 11) was reduced on the 13th (13 ± 0.29 exposed vs. 15 ± 0.15 cm control, *p* = 0.001) and 20th days of life (17 ± 0.39 exposed vs. 19 ± 0.35 cm control, *p* = 0.001) compared to the control animals (*n* = 11) (Figure 4). On the 27th day of life, this difference was no longer statistically significant and the animals in the SS2 group had a comparable craniocaudal length to that of the control group (23 ± 0.47 vs. 24 ± 0.29 cm control, *p* = 0.293) (Figure 4).

When analyzing the exposed groups, SS1 (*n* = 10) and SS2 (*n* = 11), at different timepoints, we observed that the craniocaudal lengths of the offspring were not statistically different at the 13th (*p* = 0.465), 20th (*p* = 0.508), or 27th days of life (*p* = 0.988) (Figure 4).

### 3.3. Histological and Immunohistochemical Analysis of Cytokines in Thyroid Glands of Offspring of SS1 Group (600 m from CPC)

**Figure 5:**
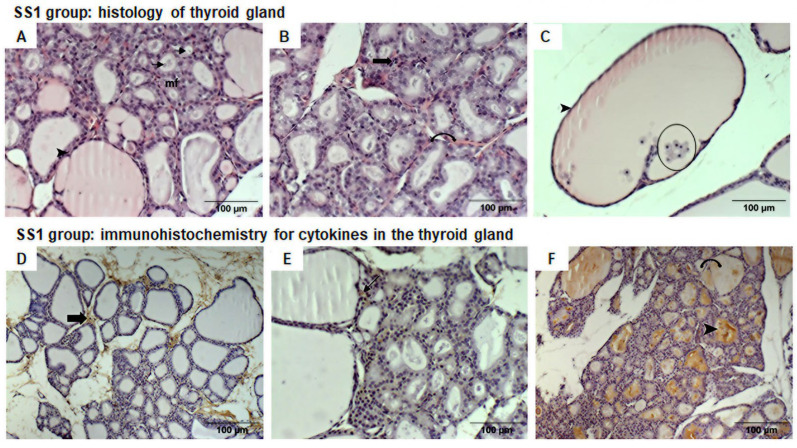
Photomicrographs (200× magnification) of HE-stained thyroid gland slices from the offspring of the SS1 group (**A**–**C**). (**A**) reveals various-sized follicles with predominance of microfollicles (mf), where some lack colloid (arrow). In large follicles, flat follicular cells are evident, rendering them squamous (arrow head). (**B**) shows an abnormal amount of cells among follicles (thick arrow) and tissue congestion (curved arrow) is evident. (**C**) demonstrates macrofollicles containing inflammatory cells, predominantly macrophages (circle) and squamous follicle cells (arrow head). Photomicrographs (**D**–**F**) display thyroid glands immunohistochemically analyzed for cytokines. (**D**) IL-6 displays weak labeling in follicular cells and connective tissue between follicles (thick arrow). (**E**) IL-10 exhibits labeling in the cytoplasm of some squamous follicular cells (arrow). (**F**) TNFα has weak chestnut staining (curved arrow) and strong labeling in follicles with vesicles (arrow head).

In the SS1 group, the thyroid glands featured various-sized follicles, including some of normal size with few macrofollicles, although most were microfollicles, some of which were so small that they lacked interior colloid (Figure 5A).

The follicles included cuboidal follicle cells, but many featured larger follicles, with flat (squamous) cells indicating variation in functional activity. In addition, most follicles were irregularly shaped and, among the squamous follicular cells, some inflammatory cells (leukocytes, predominantly macrophages) were immersed in colloid (Figure 5B,C). The colloid content of some follicles was paler, with vesicles evident.

One animal in this group had a macrofollicle filled with inflammatory cells (macrophages, mast cells, plasmocytes, and eosinophils), together with intense mitotic activity in the follicular cells.

There was an abnormal number of cells among the follicles and the loose connective tissue between follicles had abundant thin collagen fibers, making the follicles more distal from each other. The blood vessels appeared to be congested with red blood cells, suggesting generalized vasodilation.

In this group, we observed slight immunohistochemical staining for IL-6; however, this was significantly higher than that in the control group (78.59 ± 3.6 pixels/µm^2^ vs. 62.17 ± 5.5 pixels/µm^2^ control group, *p* = 0.042), displaying only connective tissue between follicles. IL-10 labeling was also significantly greater in the SS1 group compared to the control group (82.84 ± 3.2 pixels/µm^2^ vs. 68.17 ± 4.0 pixels/µm^2^ control group, *p* = 0.013), and in contrast, IL-10 staining was evident in some squamous follicles (Figure 5D,E).

Immunohistochemistry for TNFα revealed weak chestnut staining in follicles. Despite this, significantly greater TNFα labeling occurred in the area of follicles with vesicles compared to the control group (105.88 ± 7.4 pixels/µm^2^ vs. 70.08 ± 7.5 pixels/µm^2^ control group, *p* = 0.002) (Figure 5F).

### 3.4. Histological and Immunohistochemical Analysis of Cytokines in Thyroid Glands of Offspring of SS2 Group (1000 m from CPC)

**Figure 6:**
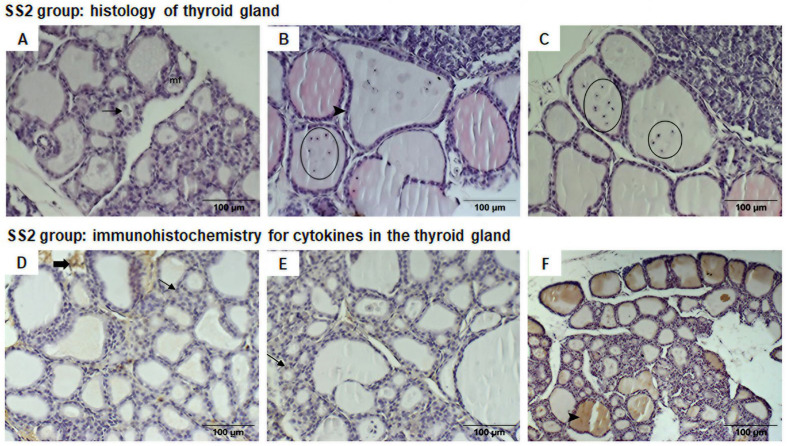
Photomicrographs (200× amplification) of HE-stained thyroid gland slices from the offspring of the SS2 group (**A**–**C**). (**A**) reveals irregularly shaped follicles with many microfollicles (mf), some of which lack colloid (arrow). (**B**) shows flat, squamous follicular cells (arrow head), macrofollicles with irregular shape, and inflammatory cells (macrophages, mast cells and plasmocytes) immersed in colloid (circles). (**C**) reveals inflammatory cells immersed in colloid (circle) and colloid with pale staining. (**D**–**F**) are photomicrographs of the immunohistochemically stained thyroid gland. (**D**) IL-6 and (**E**) IL-10 display weak staining in connective tissue (thick arrow) and in cytoplasm of squamous follicular cells (arrow). (**F**) TNF shows chestnut staining within all follicles (arrow head).

In the SS2 group, the thyroid glands also exhibited follicles of varying sizes, some of normal size, with the presence of some macrofollicles, but these were mostly microfollicles, many of which did not contain any colloid. The follicles included cuboidal follicle cells; nevertheless, the follicular cells in many follicles, particularly macrofollicles, were flat (squamous). In addition, some follicles were irregularly shaped, and some inflammatory cells were immersed in the colloid, including macrophages, mast cells, and numerous plasmocytes (Figure 6A–C). The colloid content of some follicles was paler, with vesicles evident.

There was an abnormal amount of cells between the follicles, and leukocyte infiltrate cell clusters were evident in some regions. Thin collagen fibers were visible in the loose connective tissue between follicles. Blood vessels were congested with red blood cells, suggesting generalized vasodilation.

In this group, mild staining in the connective tissue was seen for IL-6 and IL-10. Follicles with squamous cells exhibited significantly increased IL-6 labeling compared to the control group (78.94 ± 4.4 pixels/µm^2^ vs. 62.17 ± 5.5 pixels/µm^2^ control group, *p* = 0.040) as well as significantly greater IL-10 labeling in the cytoplasm of some of these cells (84.37 ± 2.5 pixels/µm^2^ vs. 68.17 ± 4.0 pixels/µm^2^ control group, *p* = 0.006) (Figure 6D,E).

Immunohistochemistry for TNF-α showed chestnut staining within all follicles, and significantly higher TNF-α labeling than in the control group (105.32 ± 4.6 pixels/µm^2^ vs. 70.08 ± 7.5 pixels/µm^2^ control group, *p* = 0.002) (Figure 6F).

**Figure 7:**
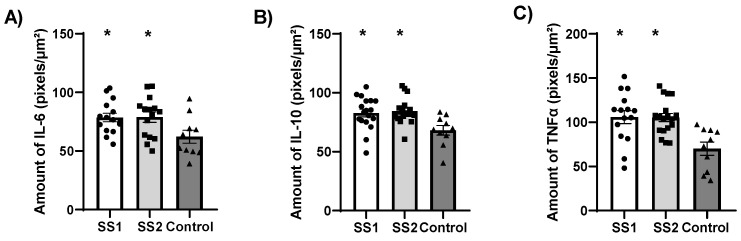
Quantitative analysis of immunohistochemical labeling of IL-6 (**A**), IL-10 (**B**), and TNF-α (**C**) (pixels/µm^2^). Data are presented as mean ± SEM. Both exposed groups exhibited significantly higher cytokine expression compared to controls (* *p* < 0.05), with no significant difference between SS1 and SS2. (One-way ANOVA followed by Tukey’s post hoc test).

No significant difference was observed in the IL-6, IL-10, and TNFα labeling between the SS1 and SS2 groups (Figure 7).

### 3.5. Histological and Immunohistochemical Analysis of Thyroid Glands from Offspring of Control Group

**Figure 8:**
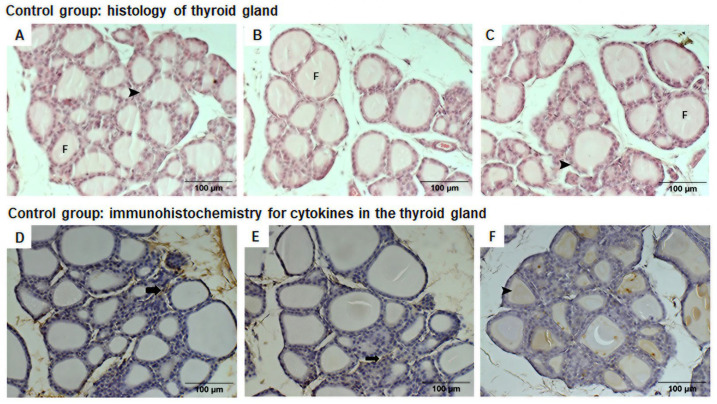
Photomicrographs (200× amplification) of HE-stained thyroid glands from control animals (**A**–**C**) revealing regular-shaped follicles (F) and cuboidal follicular cells (arrow head). Photomicrographs of immunohistochemically stained thyroid glands from control animals (**D**–**F**). (**D**) IL-6 and (**E**) IL-10 display weak staining in loose connective tissue between follicles (thick arrow). (**F**) TNF-α exhibits pale chestnut staining within follicles (arrow head).

The control group had thyroid glands with regular-sized follicles, i.e., no major differences in follicle size were observed, and there were no microfollicles. The follicles contained colloid, which exhibited eosinophil staining, indicating protein concentrated in this region (Figure 8A–C). Loose connective tissue with blood vessels and sparse, delicate collagen fibers were evident between follicles. We did not observe any inflammatory cells (polymorphonuclear or mononuclear) in this group’s thyroid glands (Figure 8A–C).

Weak immunohistochemical staining in the connective tissue between follicles was observed for IL-6 and IL-10 (Figure 8D,E). Immunohistochemistry for TNF-α revealed pale chestnut staining within follicles (Figure 8F).

## 4. Discussion

The findings of the present study reveal that the distance from the CPC contributed to the lower body weight and shorter craniocaudal length of the offspring born at the SS1 and SS2 locations, which were exposed to pollutants released by the CPC, in comparison to the control animals kept at a location far from the CPC. The results for the exposed groups (SS1 and SS2) at the different timepoints revealed that the SS1 group’s descendants had a lower body weight after birth on the 6th, 13th, and 20th day of life compared to the SS2 group’s descendants.

The health impacts caused by industrial emissions in the region surrounding the CPC have previously been demonstrated [11,12,13,14] and Air Quality Stations (AQS) have recorded pollutant levels that consistently exceed the limits established for air quality in Brazil [39,40]. Studies have compared the periods 2017–2020 and 2021–2022, drawing on the official air quality monitoring network’s reported cases of PM10, benzene, and toluene release in order to lower the proportion of uncontrolled emissions [41]. Close-field simulations have also been conducted to assess the distribution of industrial pollutants, applying the Gaussian plume model AERMOD (a steady-state plume model), estimating the VOC and PM10 concentrations to which the population was exposed in the vicinity of the CPC—the same region where the exposed animals in the present study were placed in 2019.

The results of the study by Coelho et al. [41], comparing data from the past 4 years, showed a 2-fold increase in mean concentrations of PM10, benzene, and toluene, reaching maximum values of 174 μg.m^−3^ (PM10), 79.1 μg.m^−3^ (benzene), and 58.7 μg.m^−3^ (toluene) during this period. The AERMOD showed that VOC plumes had the potential to reach large swathes of the cities of Maua and Santo Andre, SP, and affect the health of over 1 million residents.

The results of the same study [41] corroborate the findings of an investigation into PM10, nitrogen dioxide (NO_2_), sulfur dioxide (SO_2_), carbon monoxide (CO), and VOCs [14] in the vicinity of the CPC, which showed high levels of both PM10 and NO_2_ compared to another region far from the CPC.

The very small diameter of PM is thought to make it more harmful, because this enables it to penetrate deep into the lungs, lodging in bronchioles and alveoli within the pulmonary parenchyma. Thus, in many regions, the current focus is on measuring and regulating PM less than 2.5 mm (PM 2.5) in diameter. According to the World Health Organization (WHO), the annual arithmetic average for PM 2.5 should not exceed 10 μg/m^3^, whereas the 24-h period for the PM 2.5 average should be below 25 μg/m^3^ [42].

Since the 1990s, the Environmental Company/Environmental Sanitation Technology Company of Sao Paulo State (CETESB) and AQS have been monitoring O_3_, NO_2_, SO_2_, inhalable particles (PM10), fine PM (PM 2.5), and CO levels in the MetASP [40]. PM10 and PM 2.5 levels observed worldwide and in the MetASP have exceeded the WHO’s recommended limits [42]. Moreover, human exposure to these particles was rated as one of the ten main environmental risk factors to health [43].

During fetal development, the routes of entry of atmospheric pollutants into the fetus include inhalation by the mother. The particles themselves can have adverse effects on fetal development, as well as their associated chemical products, which can enter the bloodstream and impact the placenta and fetus directly, or indirectly via pollution that induces inflammation and impacts maternal circulation. In pregnant mothers, exposure to air pollution has been associated with a plethora of adverse effects, including inflammation and oxidative stress [44]. Air pollution has been associated with changes in blood flow between the umbilical cord and placenta and with reducing oxygen levels, and has also been implicated as a mechanism underlying fetal growth retardation [45].

The present study’s results revealed that the offspring of rats exposed (SS1 and SS2 groups) to high levels of PM 2.5 in the vicinity of the CPC were found to have shorter craniocaudal lengths and lower body weights [40].

Previous studies have shown the impact of atmospheric PM 2.5 pollution during the perinatal period [37,46]. For instance, in 2017, Smith et al. [47] found that exposure to levels of PM 2.5 > 13.8 μg.m^−3^ during pregnancy was directly associated with slower fetal growth rates. They [47] examined 540,365 births in London and found that prolonged exposure to NO_2_ and PM 2.5 was associated with an increased risk of low birth weight. In addition, exposure to PM 2.5 and PM10 during the third trimester was associated with an adverse effect on fetal growth rate [47].

The impact of PM 2.5 on fetal development has also been shown through a review of 124 global studies, which estimated that atmospheric PM 2.5 pollution was associated with a 7.5–13 g reduction in the birth weight of infants born in the United Kingdom [45,46].

Smith et al. [47] also found that exposure to ozone (O_3_) was associated with greater risk of preterm birth.

In 2017, the CETESB began monitoring timepoints at which benzene and toluene were released at the Capuava AQS, the only station that monitors these compounds in the area, representing a reference for industrial emissions in the region [40].

Health concerns have been raised regarding different sources of emissions, including oil refining and petrochemical industries. The possibility of metals being present in PM is a key issue [48].

Heavy metals are toxic chemical elements that can disperse in water, soil, and air, and are characterized by their propensity to react with other chemical substances and inability to be broken down or destroyed, and are thus defined as bioaccumulative [49].

The bioaccumulation of heavy metals leads to a host of deleterious effects in a variety of bodily tissues and organs. Increased production of reactive oxygen species (ROS) and oxidative injury have been implicated as ramifications of heavy metal toxicity [50], which can disrupt cellular events, including cell growth, proliferation, differentiation, damage repair processes, and apoptosis. Heavy metals can also promote epigenetic changes, which can influence gene expression [49].

Biomonitoring of air pollution in the city of Santo Andre has revealed the highest concentrations of aluminum (Al), barium (Ba), calcium (Ca), chlorine (Cl), Iron (Fe), potassium (K), magnesium (Mg), manganese (Mn), phosphorus (P), sulfur (S), strontium (Sr), Zn, Cd, Ni, Pb, and V [51] in the vicinity of the CPC, and here, concentrations have not decreased significantly during the period in which this study was conducted [51].

The findings of the current study might be at least partially underpinned by the effects of fetal toxicity of V reported in the literature [52,53]. Previous studies have shown that administration of V_2_O_5_ leads to a decrease in fetus weight and delayed ossification [53].

A study by Paternain et al. [54], in which VOSO_4_ was administered daily by gavage to pregnant Swiss mice, has also reported embryo and fetal toxicity of this element.

Recent studies have shown that prenatal exposure to PM 2.5 can lead to impaired thyroid function in neonates, while evidence suggests that the effect of this pollutant can be partially mediated by the maternal thyroid [21].

The thyroid hormones 3,5,3′,5′-tetraiodothyronine, or thyroxin (T4), and 3,5,3′-triiodothyronine (T3) contain iodine atoms as part of their structure, and their synthesis occurs in unique structures called thyroid follicles, necessary for fetal development and growth [24,55].

Thyroid hormones are essential for fetal growth and development, and a reduction in the 10–90 percentage ratio of free thyroid (FT4) levels in umbilical cord blood has been associated with a 56 g decrease in mean birth weight (95% CI: −90, −23). Moreover, PM 2.5 air pollution has been associated with changes in fetal thyroid hormone levels, which can contribute to low birth weight [19]. Free thyroid (FT4) levels are inversely associated with exposure to PM 2.5 and NO_2_, while exposure to PM 2.5 has also been associated with a higher risk of maternal hypothyroxinemia [56]. Studies carried out in the vicinity of the CPC have shown high levels of both PM 2.5 [40] and NO_2_ [14].

The present study revealed histological changes in the thyroid tissues of offspring exposed to the CPC (SS1 and SS2) that were not seen in the control animals. Irregularly sized thyroid follicles were found, where larger follicles had follicular cells with flat nuclei, some of which contained inflammatory cells such as macrophages, mast cells, and plasmocytes immersed in the colloid. The size variation in the follicles and shape alterations in the follicular cells suggest changes in the functional activity of the gland. In addition, an increase in the connective tissue between follicles compared to controls was also evident. Although few studies have shown changes in the thyroid gland after exposure to pollution, the investigation performed by Mohamed and El-Twab [57] also found alterations in the shape of cells and size of follicles in thyroids exposed to potassium dichromate. The differences in follicle size and increase in connective tissue, together with the changes mentioned, are suggestive of colloid goiter.

The presence of inflammatory cells, such as macrophages, mast cells, plasmocytes, and eosinophils, within the follicles of the thyroid suggest a local immune response, indicating an inflammatory reaction in response to cell injury or stress. This theory is reinforced by the results found for the inflammatory cytokines IL-6, IL-10, and TNF-α. TNF-α labeling within follicles can be associated with the presence of inflammatory cells in this region. IL-6 and IL-10 exhibited more diffuse staining both in the connective tissue between follicles and the cytoplasm of some squamous cells of large follicles. The distribution of these cytokines may indicate distinct patterns of an inflammatory response in different regions of the thyroid. Moreover, the exposed groups had more dilated blood vessels, showing generalized vasodilation. This suggests changes in the vascularization of the gland, possibly as a consequence of inflammation, and could be associated with mast cells seen in the follicles of some animals.

The evaluation of the proportions and correlations between benzene, toluene, ethylbenzene, and xylenes (BTEX), all aromatic hydrocarbons that constitute a fraction of VOCs, confirmed the environmental impact of the CPC [5]. Other studies [14] have shown higher levels of VOCs close to the CPC compared to areas further away from this industrial area.

The interactions between the different combined chemical elements, including several heavy metals, can be synergistic or antagonistic. Those with other chemical compounds, such as organic and inorganic substances, can also potentially disrupt thyroid regulation [58].

In this study, we can hypothesize that an impairment in the production of thyroid hormones could affect the development of the rat pups. Interrelationships between growth hormone and thyroid hormones in the hypothalamic, pituitary, and peripheral tissues have already been demonstrated [59,60]. Thus, the exposure of the rat pups to air pollutants via the maternal bloodstream during intrauterine development likely evoked an inflammatory response that could have affected thyroid hormone production and/or secretion, and consequently, may have impaired the synthesis and secretion of growth hormone, leading to the lower craniocaudal length and reduced body weight observed in these pups compared to the control animals. Despite these pups showing these impairments, their craniocaudal length and body weight became close to those of the control group 27 days after birth. Kurashige et al. [61] have demonstrated the crucial role of autophagic activity in thyrocyte survival. In our study, it is possible that autophagy was a key process that allowed the recovery of thyroid gland activity after birth in the pups exposed to different air pollutants, and consequently enabled the growth and body weight of the animals to normalize.

The WHO [62] estimates that, annually, over 20 million neonates are born with low birth weight and a further 15 million are preterm (<37 weeks of gestational age) and, according to the Office of National Statistics 2022 (ONS) [35], this represents an important factor contributing to infant mortality.

Low birth weight and preterm birth are known risk factors for mortality in early life and for morbidity during adulthood [63].

Wang et al. [64] pointed out that petroleum hydrocarbons as well as heavy metals can cause contamination in petrochemical industrial areas, and urged for more attention to the health risks posed to populations in these areas.

Globally, an estimated 2.8 million low-weight births and 5.9 million preterm births could be avoided if exposure to PM 2.5 during pregnancy was kept to within the theoretically safe minimum thresholds of exposure of 2.4–5.9 μg m^−3^ [45].

A global assessment by Malley et al. [65] examined the total number of preterm births associated with maternal exposure to PM 2.5. The authors found that 18% of all preterm births worldwide were associated with an average annual exposure to PM 2.5 exceeding 10 μg.m^−3^. According to Ghosh et al. [45], the average reduction in gestational age attributable to PM 2.5 was 0.4–07 weeks (approximately 3–5 days).

We did not measure the levels of thyroid hormone (T3 and T4), thyroid-stimulating hormone (TSH), anti-thyroid antibodies—such as anti-thyroid peroxidase (A-TPO) and anti-thyroglobulin (A-Tg) antibodies—or blood growth hormone (GH) in the animals assessed. Despite that we can hypothesize that the reduced craniocaudal length at birth and lower body weight in offsprings exposed to air pollutants in CPC surrounding area (SS1 and SS2) could be due to the impairment in the production of thyroid hormones, which are essential for body development. Such hypothesis could be underpinned by our findings in the current study, which showed the thyroid gland with evident morphological alterations and inflammatory labeling. In addition, we did not measure the temperature and humidity in the area where the animals were studied, both of which are limitations of this study. It is important to emphasize that, in the present study, offspring were not stratified by biological sex and were exposed to the polluted environment exclusively during the first 27 days of life, a period corresponding to early postnatal development in rodents characterized by limited functional sexual dimorphism. Evidence suggests that, during the neonatal and pre-pubertal stages, many systemic responses to environmental exposure, including somatic growth, metabolic maturation, inflammatory signaling, and neural development, are less sex-dependent than in the post-pubertal and adult stages, as full activation of the hypothalamic–pituitary–gonadal axis has not yet occurred [66,67].

Sex-specific differences in responses to airborne pollutants, heavy metals, and particulate matter tend to become more pronounced after puberty, when sex hormones exert stronger regulatory control over inflammatory, metabolic, and neuroendocrine pathways. Therefore, the early developmental window examined here likely reduced the magnitude of sex-dependent variability in the primary outcomes evaluated. Additionally, experimental studies investigating early-life environmental exposure commonly include mixed-sex groups when the focus is on global developmental endpoints, particularly during critical windows of vulnerability, as the inclusion of both sexes may enhance translational relevance despite potentially increasing variability [68,69].

Nevertheless, biological sex remains a potential modifier of environmental susceptibility, and the absence of a sex-stratified analysis may have limited the detection of subtle sex-specific effects. Future investigations incorporating sex-based stratification and extended exposure periods will be important for clarifying interactions among sex, developmental timing, and environmental pollutant exposure. Despite we have performed direct pollutant measurements, which precludes quantification of individual inhaled dose and does not allow discrimination of the relative contribution of specific contaminant classes (e.g., PM_2_._5_ versus metals or VOCs), the results of this animal study can serve as a warning regarding the need to improve the quality of ambient air and implement measures reducing atmospheric pollution so that children do not suffer the consequences of atmospheric pollutant contamination. These steps can prevent children with low birth weight and developmental delay from being susceptible to infant mortality and can also avoid the deaths of preterm neonates. Therefore, developing techniques for removing these environmental contaminants to reduce atmospheric pollution is paramount.

## 5. Conclusions

The results of this study indicate that proximity of rats to the CPC was a determining factor in the development of histological abnormalities in the thyroid glands of their offspring, with an increase in inflammatory cytokine markers observed. In addition, the offspring born near the CPC had a lower birth weight and shorter craniocaudal length compared to the animals in the control group.

## Data Availability

The individual data generated in the present study and additional details of the materials will be available upon request to the corresponding author.

## Abbreviations

The following abbreviations are used in this manuscript:

TNFα	Tumor Necrosis Factor alpha
IL-6	Interleukin 6
IL-10	Interleukin 10
CPC	Capuava Petrochemical Complex
MetASP	Metropolitan Area of Sao Paulo
SS1	600 m from the CPC
SS2	approximately 1000 m from the CPC
VOCs	Volatile Organic Compounds
PM	Particulate Material
SOA	Secondary Organic Aerosol
BTEX	Benzene, Toluene, Ethylbenzene, and Xylene
WHO	World Health Organization
O_3_	Ozone
LPG	Liquefied Petroleum Gas
AQS	Capuava Air Quality Stations
AITD	Autoimmune Thyroid Disease
PH	Primary Hypothyroidism
V	Vanadium
Ni	Nickel
EPA	U.S. Environmental Protection Agency
Pb	Lead
CNS	Central Nervous System
Zn	Zinc
Cu	Copper
Cd	Cadmium
H&E	Hematoxylin–Eosin
LEMT	Laboratory of Molecular and Translational Endocrinology
AERMOD	Gaussian plume model (steady-state plume model)
NO_2_	Nitrogen Dioxide
SO_2_	Sulfur Dioxide
CO	Carbon Monoxide
CETESB	Environmental Company/Environmental Sanitation Company of Sao Paulo State
ROS	Reactive Oxygen Species
ONS	Office of National Statistics
